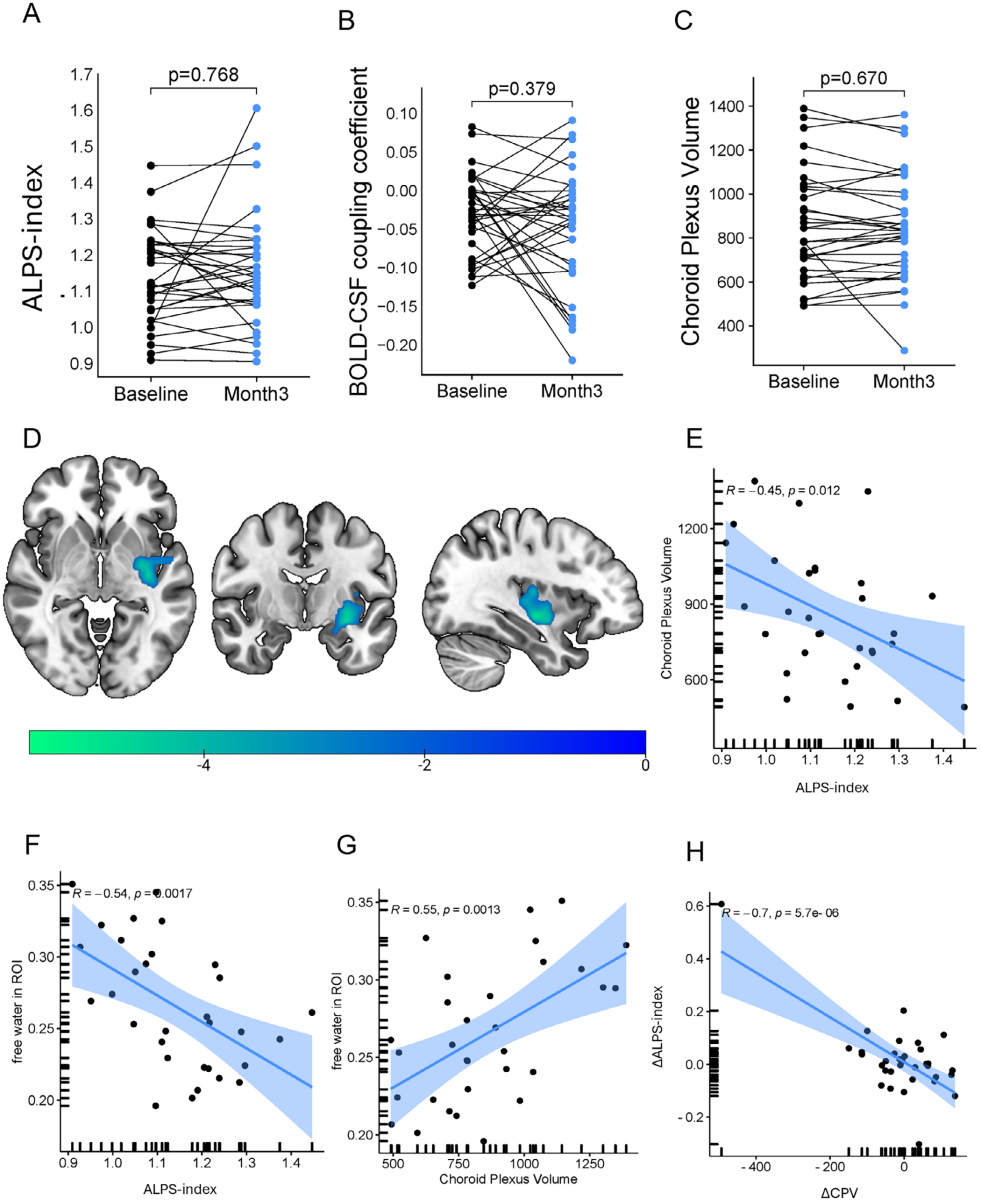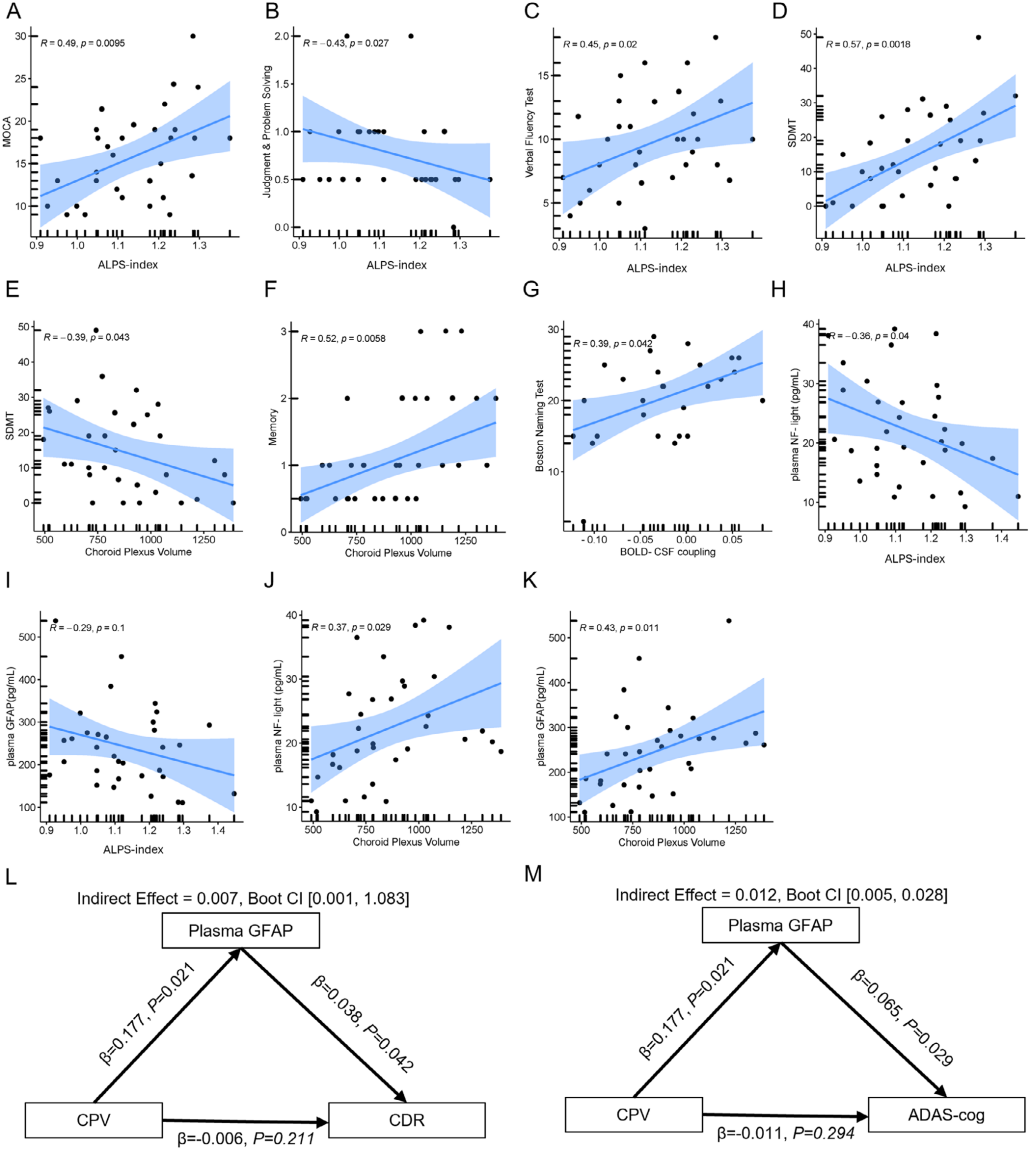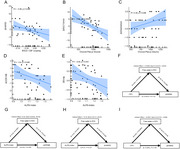# Lecanemab therapy Leads to Glymphatic Function Maintenance and Free Water Homeostasis in Alzheimer’s Disease: Evidence from a Multidimensional Real‐World Study

**DOI:** 10.1002/alz70861_108148

**Published:** 2025-12-23

**Authors:** Ruinan Shen, Huizhu Zhong, Qiao Wei, Chao Gao, Xiaoyan Li, Lingyun Chen, Yonghua Tang, Jun Liu, Wenyan Kang

**Affiliations:** ^1^ Ruijin Hospital affiliated with Shanghai Jiao Tong University School of Medicine, Shanghai Singapore; ^2^ Ruijin Hospotal, Shanghai JiaoTong University, School of Medicine, Shanghai China; ^3^ Ruijin Hospital, Shanghai Jiao Tong University School of Medicine, Shanghai China; ^4^ Department of Neurology, Hainan Branch, Ruijin Hospital, Shanghai Jiao Tong University School of Medicine, Shanghai, Shanghai China; ^5^ Medical department, Eisai China Inc, Shanghai China; ^6^ Department of Neurology and Institute of Neurology, Ruijin Hospital affiliated to the Shanghai Jiaotong University School of Medicine, Shanghai, Shanghai China; ^7^ Department of Neurology, Institute of Neurology, Ruijin Hospital, Shanghai Jiao Tong University School of Medicine, Shanghai, Shanghai China

## Abstract

**Background:**

The glymphatic system’s role in mediating anti‐amyloid therapy efficacy remains underexplored. This study investigates the effects of Lecanemab on glymphatic function, free water distribution, and cognition performance in Alzheimer’s disease.

**Method:**

In this real‐world study, 33 amyloid‐PET‐positive participants (≤4 microbleeds) received Lecanemab who completed 3‐month follow‐up. Assessments included cognitive performance, plasma biomarkers (NfL, GFAP), and neuroimaging (ALPS index, BOLD‐CSF coupling, choroid plexus volume [CPV], free water [FW] mapping). Mediation analyses evaluated glymphatic‐cognitive relationships.

**Result:**

Glymphatic parameters showed no alteration after 3‐month Lecanemab treatment. FW decreased in left temporal‐limbic regions post‐Lecanemab (*p* <0.05). Glymphatic parameters correlated with cognition performance and plasma biomarkers (NfL and GFAP). Baseline glymphatic metrics correlated with cognitive improvement, with FW mediating glymphatic effects on ΔMMSE and ΔHAMD.

**Conclusion:**

Short‐term Lecanemab stabilizes glymphatic function, reduces FW of temporo‐limbic region, and associates with cognitive benefits partially mediated by FW. Baseline glymphatic integrity may predict a therapeutic response, highlighting its role in anti‐amyloid therapies.